# Assessing visuo-constructive functions in patients with subjective cognitive decline, mild cognitive impairment and Alzheimer’s disease with the Vienna Visuo-Constructional Test 3.0 (VVT 3.0)

**DOI:** 10.1007/s40211-021-00385-x

**Published:** 2021-01-28

**Authors:** Noel Valencia, Johann Lehrner

**Affiliations:** Department of Neurology, Medical University of Vienna, Währinger Gürtel 18–20, 1097 Vienna, Austria

**Keywords:** Visuo-constructive functions, Subjective cognitive decline, Mild cognitive impairment, Alzheimer’s disease, Neurocognitive disorder, Visuo-konstruktive Fähigkeiten, Subjektive kognitive Störung, Leichte kognitive Störung, Alzheimer Krankheit, Neurokognitive Störung

## Abstract

**Background:**

Visuo-Constructive functions have considerable potential for the early diagnosis and monitoring of disease progression in Alzheimer’s disease.

**Objectives:**

Using the Vienna Visuo-Constructional Test 3.0 (VVT 3.0), we measured visuo-constructive functions in subjective cognitive decline (SCD), mild cognitive impairment (MCI), Alzheimer’s disease (AD), and healthy controls to determine whether VVT performance can be used to distinguish these groups.

**Materials and methods:**

Data of 671 participants was analyzed comparing scores across diagnostic groups and exploring associations with relevant clinical variables. Predictive validity was assessed using Receiver Operator Characteristic curves and multinomial logistic regression analysis.

**Results:**

We found significant differences between AD and the other groups. Identification of cases suffering from visuo-constructive impairment was possible using VVT scores, but these did not permit classification into diagnostic subgroups.

**Conclusions:**

In summary, VVT scores are useful indicators for visuo-constructive impairment but face challenges when attempting to discriminate between several diagnostic groups.

## Abstract (de)

**Hintergrund:**

Visuokonstruktive Funktionen bieten ein Potenzial für die frühzeitige Diagnose und Überwachung des Krankheitsverlaufs bei der Alzheimer-Krankheit.

**Ziele:**

Der Vienna Visuo-Constructional Test 3.0 (VVT 3.0) wurde verwendet, um visuokonstruktive Funktionen bei subjektivem kognitivem Rückgang (SCD), leichter kognitiver Beeinträchtigung (MCI), Alzheimer-Krankheit (AD) und gesunden Kontrollen zu untersuchen.

**Materialien und Methoden:**

Die Daten von 671 Teilnehmern wurden analysiert, indem die Ergebnisse über diagnostische Gruppen hinweg verglichen und Assoziationen mit relevanten klinischen Variablen untersucht wurden. Die prädiktive Validität wurde unter Verwendung von Receiver Operator Kurven und einer multinomialen logistischen Regressionsanalyse bewertet.

**Ergebnisse:**

Wir fanden signifikante Unterschiede zwischen der Alzheimer-Gruppe und den anderen Gruppen.

**Schlussfolgerungen:**

Zusammenfassend sind VVT-Scores nützliche Indikatoren für visuell-konstruktive Beeinträchtigungen.

## Keypoints

Assessing visuo-constructive functions is a suitable approach for the early diagnosis of neurodegenerative conditions.

VVT scores are useful indicatiors for identifying patients suffering from Alzheimer’s disease and related visuo-constructive impairment.

It is not possible to classify patients in all stages of Alzheimer’s disease progression, namely SCD, MCI, AD and healthy controls into these specific groups reliably using only VVT scores.

## Introduction

The World Health Organization [1] (WHO) has issued a call to make dementia a public health priority in view of the world’s aging population and the overwhelming impact this condition has on patients, families and societies. One of the key areas of concern that this organization identified is early diagnosis, as the early stages of dementia still tend to be overlooked despite numerous research results [2, 3] which suggest that the onset of disease-related brain alterations substantially precedes the manifestations of symptoms. We thus require reliable and valid instruments that enable us to detect the mentioned changes as early as possible and identify the level of disease progression in patients.

According to the WHO [1], dementia is a syndrome caused by progressive brain disease, which features disturbances in several higher cortical functions. The most common of these underlying diseases is Alzheimer’s Disease (AD) [1]. One common theory [4] suggests that dementia progresses in several stages. The early stage is characterized by subjective cognitive decline (SCD), which progresses to mild cognitive impairment (MCI) and eventually to dementia. During the transitional SCD phase, patients notice a subjective deterioration in cognitive functioning but display inconspicuous neuropsychological test performance when compared to a sociodemographically-adjusted norm [4]. This changes, however, when patients enter the MCI stage, in which an objective decline in performance can be detected reliably [3, 5]. Eventually, cognitive impairment reflecting the neurodegenerative processes becomes severe enough to cause deficits in social and occupational functioning, thus meriting the diagnosis of dementia [5].

Many researchers have reported on the value of measuring visuo-constructive functions in neuropsychological assessment to identify subjects suffering from neurocognitive disorders [6–8]. Two recent reviews, for instance, independently identified visuo-constructive functions as one important cognitive domain for early detection of dementia [9] and the monitoring of disease progression from MCI to dementia [10]. In addition, the fact that visuo-constructive deficits can become apparent quite early in the course of dementia [8, 9, 11] increases the importance of assessing this cognitive domain for early diagnosis of dementia. This early decline during visuo-constructive tasks presumably reflects underlying neurodegenerative changes occurring especially in parts of the parietal lobes [9, 12]. Such changes have been detected during all stages of AD, including the early ones [13]. Ocurrences related to broader visuo-spatial functioning, such as patients getting lost or misplacing objects, are often among the striking features first noticed in early AD [14]. However, patients can also suffer from more subtle deficits, such as difficulties in reading or in form and color perception, which may ultimately progress to visual agnosia [15]. Different assessment modes of visuo-constructive functions have been proposed based either on free-drawing (e.g. clock-drawing [16]) or copying tasks [17]. Copying tasks can be divided into those using relatively simple geometrical figures such as circles and squares (e.g. in the test battery of the Consortium to Establish a Registry for Alzheimer’s disease (CERAD) [18]), more complex figures like a three-dimensional cube (e.g. in Alzheimer’s Disease Assessment Scale [19]) and overlapping pentagons (e.g. in Mini Mental State Examination (MMSE) [20]) or very complex figures (e.g. The Rey-Osterrieth complex figure test (ROCF) [21, 22]).

In view of the above, many clinical institutes have published diagnostic guidelines recommending comprehensive evaluation of several cognitive domains including visuo-constructive functions. The Diagnostic and Statistical Manual of Mental Disorders (DSM-5) [23], for example, lists perceptual-motor functioning (including the subdimension visuo-constructional reasoning) as a key domain for the diagnosis of neurocognitive disorders. The National Institute on Aging–Alzheimer’s Association also includes this domain in its guidelines for neuropathological assessment of mild cognitive impairment in Alzheimer’s disease [24].

### The Vienna Visuo-Constructional Test (VVT)^1^

Taking the factors mentioned above into account, the VVT was developed at the Medical University of Vienna as a new instrument for assessing visuo-constructive functions. Patients are instructed to copy a clock (a task similarly used e.g. in the Montreal Cognitive Assessment (MoCA) [25]), two overlapping pentagons (as in MMSE [20]), and a three-dimensional cube (as in ADAS-Cog [19]). The Clockdrawing Test is a well-established screening instrument in the assessment of AD (e.g. described in review by Pinto, Peters [16]). Notably, in the VVT a clock-copying task is included instead of a free clock drawing in order to provide a purer measure of visuo-constructive ability that is not confounded by additional abilities required for the free drawing task, such as planning or abstract thinking [16], which tend to show more frontal than parietal involvement [14]. Similarly, pentagon-copying tasks have been shown to be useful in the discrimination of AD and dementias of other subtypes [26], as well as between AD and HC [27], and have also shown some potential as indicators of global cognitive functioning of AD patients [28]. Finally, cube-copying is a task in which AD patients have been reliably shown to perform worse than HC [29], even during early stages of the disease [30].

The psychometric properties of the VVT have been analyzed by Lehrner et al. [7]. These authors have reported adequate internal consistency of 0.82 for HC and 0.93 for the total clinical sample, respectively, as a measure of the VVT’s reliability. Their analysis also shows **satisfactory results in analyses of discriminant validity**^1^. Two scoring versions of the VVT are available, namely a full version and a screening version which consists of fewer scoring items and can therefore be scored faster. Hereinafter, all mentions of VVT refer to the full version, which consists of 98 scoring items (32 for the copied clock, 26 for the overlapping pentagons, and 40 for the cube). Patients’ copies of figures are scored for those 98 items that have been shown to provide the best item selectivity in previous analyses [7], rating overall size, alignment, and length of individual lines, together with other criteria.

In summary, when compared with three individual copying tasks, the VVT provides a more thorough assessment of visuo-constructive functions since it combines the information of the established figures with an exact and extensive scoring system and partially eliminates the executive component required for other tasks (e.g. free clock drawing or ROCF). Based on these advantages, our goal was to further assess the utility of visuo-constructive assessment using VVT scores for clinical classification of HC, SCD, MCI, and AD in the present design.

### Research questions of the present design using the VVT 3.0

As a first step in the present design, we planned to assess the VVT’s ability to detect visuo-constructive impairment by using AD group membership as an indicator, thus permitting the discrimination of AD and non-AD (HC, SCD, MCI) cases. Further on, we planned to look into VVT’s potential for a more complex classification into an impairment-free group consisting of HC and SCD, a mildly impaired group (MCI), and a strongly impaired group (AD). The purpose of this was to ascertain whether VVT scores contain enough diagnostic information to predict diagnostic group membership for the cited impairment levels. We expected HC and MCI, HC and AD, SCD and MCI, SCD and AD, and MCI and AD to differ in VVT scores, but did not expect HC and SCD to do so since SCD patients do not yet show a quantifiable decline in performance. For the same reason, we did not expect a successful discrimination between HC and SCD in the classification procedures. We also planned to confirm prior findings [31] of indistinguishable performance in figure copying between male and female participants.

In addition to the above, Lehrner et al. [7] conducted a first analysis of associations between VVT scores and age, premorbid IQ, depression and the test variables of the broader Neuropsychological Test Battery Vienna (NTBV) [32] and reported negligible-to-low correlations for all of these variables. A further goal of our design was to take a second look at these associations with our considerably larger sample. We expected to find correlations of similar size and confirm their first results.

## Materials and methods

Our analyses are based on anonymized data of mostly Austrian participants referred to the Department of Neurology at the Medical University of Vienna for assessment of cognitive functions collected between 2008 and 2020 in the course of the Vienna Conversion to Dementia Study. This design was approved by the Ethics Committee of the Medical University of Vienna and was conducted in accordance with the Declaration of Helsinki.

### Sample characteristics

A sample of 671 adult participants was the basis of our analyses. Freedom of neurological, psychiatric or any other comorbidity which could impact cognition, freedom of psychotropic medication, and a minimum of eight years of formal schooling had previously been defined as necessary inclusion criteria following the recommendations for Mayo research studies [33]. Descriptive statistics for the sample and diagnostic sub-groups can be found in Table 1. The frequencies in the diagnostic groups were distributed as follows: 77 patients suffered from SCD (11.5%), 288 patients suffered from MCI (42.9%), 228 patients suffered from AD (34%), and 78 patients in the separate HC group (11.6%). Patients were diagnosed based on a clinical interview together with a neuropsychological assessment of cognitive status using MMSE and NTBV. VVT scores were not considered for diagnosis. Specifically, classification into SCD relied on the diagnostic guidelines of Jessen et al. [34], which describe, a presentation with self-perceived cognitive decline and inconspicuous neuropsychological test performance. MCI was diagnosed according to Mayo Clinic criteria [33], which were also applied to establish healthy cognition in HC [35]. Thus, participants assigned to the MCI group showed a manifest cognitive impairment in at least one cognitive domain as assessed by the NTBV. NICDS-ADRDA [36] and DSM‑V criteria [23], including subtle disease onset together with gradual, progressive decline in memory and other cognitive domains, were applied for the diagnosis of AD. Group classifications were mutually exclusive. HC were recruited via public postings in the university clinic.

**Table 1:** Relevant Demographic and Clinical Characteristics in the Sample and its Subgroups

	Total	Male/Female	Age	Education	MMSE	VVT	VVT score different* from	VVT score not different* from
	*N*	*n*	Mean (SD)	Mean (SD)	Median (IQR)	Median (IQR)	(Mean rank)	(Mean rank)
*HC*	78	26/52	56 (16)	14 (4)	29 (2)	73 (16)	AD (212)	SCD (450) MCI (388)
*SCD*	77	40/37	66 (14)	13 (4)	29 (2)	75 (11)	AD (212)	HC (414) MCI (388)
*MCI*	288	144/144	68 (12)	13 (4)	28 (3)	73 (14)	AD (212)	HC (414) SCD (450)
*AD*	228	101/127	74 (8)	11 (3)	21 (4)	61 (22)	HC (414) SCD (450) MCI (388)	–
*∑*	671	311/360	68 (13)	12 (4)	27 (6)	69 (16)	–	–

Age and education in years

*VVT* Vienna Visuo-Constructional Test, *HC* healthy controls, *SCD* subjective cognitive decline, *MCI* mild cognitive impairment, *AD* Alzheimer’s disease, *MMSE* Mini Mental Status Examination, *SD* standard deviation, *IQR* interquartile range

*Significant and non-significant group comparisons in VVT scores performed with Kruskal-Wallis analyses χ2(3) = 152.28, *p* < 0.001 and Dunn’s post-hoc comparisons

### Instruments

The NTBV assesses a broad range of cognitive domains, such as psycho-motor speed, attention, language, memory, and executive functioning. It has been validated as a neuropsychological instrument on numerous occasions [32]. Before administering the NTBV in our study, the MMSE was used as a screening tool to evaluate global cognitive functioning [20]. If a patient scored below 24 points, the NTBV was administered in a shortened version with 9 instead of 13 subtests and with a reduced number of repetitions in some of the remaining subtests. If a patient scored below 15 points it was not administered at all in order to avoid overstraining severely ill patients. Depression was measured using the BDI-II (Beck Depressionsinventar II) [37] and premorbid IQ was assessed using the Wortschatztest (WST) [38].

### Statistical analyses

Our statistical analyses were conducted using IBM SPSS Statistics for Windows, version 21.0. In order to compensate for family-wise error (the accumulation of type 1 errors), we adjusted the alpha level of 0.05 using Bonferroni correction. As a result, significance for all results was established with regard to an adjusted alpha of 0.001. Non-parametric Kruskal-Wallis analyses were performed to compare the means of VVT scores in all diagnostic groups. Subsequently, we used pairwise Dunn’s post-hoc comparisons to explore differences. One-tailed testing was applied in all comparisons except HC and SCD, due to postulated disease progression. Male and female cases were compared using Mann-Whitney U Test. We first assessed predictive validity of VVT scores using Receiver Operator Characteristic (ROC) curves with AD as the positive condition, thus categorizing cases as AD and non-AD, and identified the ideal cut-off for this categorization according to the Youden Index. Both positive and negative predictive values (PPV/NPV) and positive and negative likelihood ratios (LR+/LR−) were computed. Using the multinomial logistic regression model, we obtained predictions for diagnostic group membership for each case and performed cross-tabulation analysis to compute Cohen’s Kappa as a measure of agreement between these and the observed diagnoses.

In addition, pairwise comparisons between groups based on the logistic regression model were conducted and yielded estimates for *B* coefficients and odds ratios for group classification. Finally, Spearman’s correlation analyses were applied to explore associations between the mentioned sociodemographic, emotional, cognitive and neuropsychological variables and VVT scores. Correlation size was interpreted according to the guidelines suggested by Hinkle et al. [39].

## Results

### Group comparisons

**Fig. 1:**
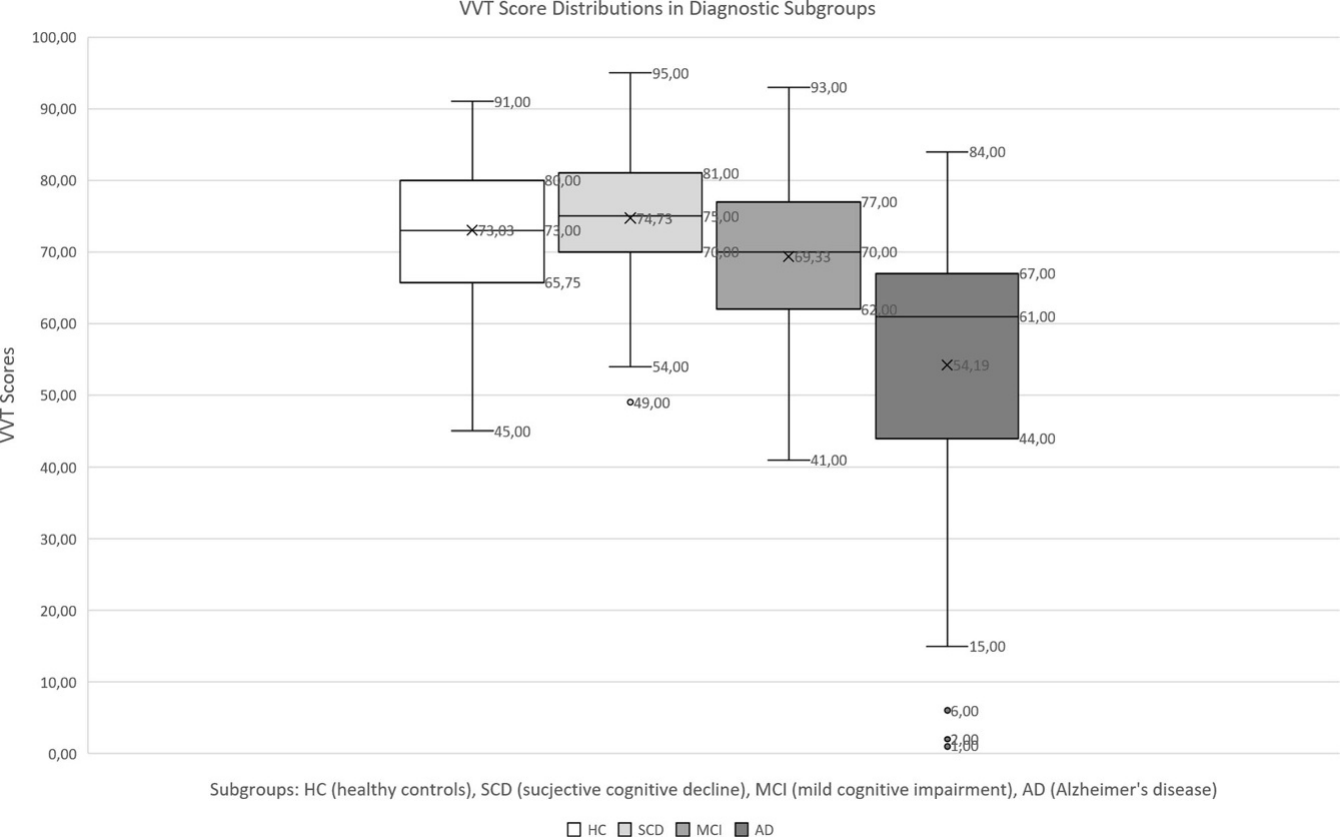
VVT Score Distributions in Diagnostic Subgroups

VVT score distributions in the diagnostic groups are displayed in Fig. 1. A comparison of VVT scores with Kruskal-Wallis analyses using diagnosis as the grouping variable yielded significant results: *χ*^*2*^(3) = 152.28, *p* < 0.001, mean ranks: HC 414, SCD 450, MCI 388, AD 212. In subsequent Dunn’s post-hoc comparisons HC was shown to differ significantly from AD (*p* < 0.001) but not from SCD (*p* = 0.129) or MCI (*p* = 0.144). SCD was again significantly different from AD (*p* < 0.001) but not MCI (*p* = 0.007). MCI also differed significantly from AD (*p* < 0.001). We also compared male and female participants using Mann-Whitney U test (*U* = 54,374, *p* = 0.521, mean ranks: male 341, female 332) and found no significant differences.

### Predictive validity of VVT scores for diagnostic group membership

Our ROC analysis using VVT scores as predictors yielded an AUC value of 0.783, 95% CI [0.747; 0.819], *p* < 0.001, at an optimal cut-off of 70 and with a sensitivity of 0.78 and a specificity of 0.64. Thus, we categorized cases with 70 or more points as non-AD and cases with less than 70 points as AD. Based on this categorization, we computed corresponding PPV of 0.53 and NPV of 0.85. Finally, LR+ was determined at 2.15 and LR− at 0.35. Taking the total sample into account and using HC as the reference category, the multinomial logistic regression model was found to be significant (*p* < 0.001) and specified with *Χ*^*2*^ = 187.36 and Pseudo-*R*^*2*^ (Nagelkerke) = 0.27. The estimates of the *B* parameters and odds ratios of the pairwise group comparisons are displayed in Table 2.

**Table 2:** Parameters of Multinomial Logistic Regression Analysis with VVT Scores as Predictors for Diagnostic Group Membership

Reference category		*B* (SE) Intercept	*B* (SE) VVT	Odds Ratio with 95% CI
HC	SCD	−1.45 (1.29)	0.02 (0.02)	1.02 [0.99; 1.06]
	MCI	2.55 (0.95)	−0.02 (0.01)	0.98 [0.96; 1.01]
	AD	7.98 (1.13)*	−0.10 (0.02)*	0.90 [0.87; 0.93]
SCD	MCI	3.98 (1.04)*	−0.04 (0.01)	0.96 [0.94;0.99]
	AD	9.94 (1.36)*	−0.13 (0.02)*	0.88 [0.85; 0.91]
MCI	AD	5.19 (0.62)*	−0.08 (0.01)*	0.92 [0.90; 0.94]

*VVT* Vienna Visuo-Constructional Test, *HC* healthy controls, *SCD* subjective cognitive decline, *MCI* mild cognitive impairment, *AD* Alzheimer’s disease, *SE* standard error

*Significance at the adjusted alpha level of 0.001

The model classified 70.7% of cases as MCI and 29.3% of cases as AD. No cases were predicted to belong to HC or SCD. Hence, 81.3% of cases with an MCI diagnosis and 53.2% of cases with an AD diagnosis were classified correctly by the regression model, but diagnostic group membership was not predicted correctly for any cases with HC or SCD diagnoses. As a result, the overall percentage of cases for which diagnostic group membership was predicted correctly was 53%. Cohen’s Kappa was determined significantly with 0.21 (*p* < 0.001).

### Correlates of VVT scores

Associations between VVT scores and relevant variables are displayed in Table 3. VVT scores displayed a moderate positive correlation with MMSE (*r* = 0.50). In the cases of premorbid IQ and depression, no relationship with VVT scores was found. A low negative correlation was found for age, and a negligible positive correlation was found for education. Many variables of the NTBV showed significant positive or negative correlations with VVT scores, but all of them were of small, barely moderate or negligible size.

**Table 3:** Spearman’s Rank Correlations Between VVT Scores and Relevant Demographic and Clinical Variables

VVT score	*r*	*p*
Age	−0.34*	<0.001
Education	0.21*	<0.001
MMSE	0.50*	<0.001
WST-IQ	0.08	0.127
BDI-II	−0.06	0.208
AKT time	−0.42*	<0.001
AKT total/time	0.44*	<0.001
Digit-symbol	0.28*	<0.001
Symbol Counting (c.I.)	−0.42*	<0.001
TMT A	−0.49*	<0.001
TMT B	−0.36*	<0.001
SWT	0.24*	<0.001
PWT	0.16	0.001
BNT	0.34*	<0.001
VSRT immediate recall	0.33*	<0.001
VSRT total recall	0.42*	<0.001
VSRT delayed recall	0.40*	<0.001
VSRT recognition	0.29*	<0.001
5‑point correct	0.27*	<0.001
5‑point perseverations	−0.18*	0.001
Stroop Test NAI‑I (time)	−0.28*	<0.001
Stroop Test NAI-III (time)	−0.29*	<0.001
Stroop Test NAI-III (total/time)	0.29*	<0.001
Stroop Test NAI-III − NAI I	−0.23*	<0.001
Labyrinth time	−0.46*	<0.001
Labyrinth total/time	0.45*	<0.001
TMT B − TMT A	−0.32*	<0.001
Interference (c.I.) time	−0.51*	<0.001
Interference (c.I) total/time	0.52*	<0.001

AKT, Digit-symbol, Symbol Counting (c.I.), TMT‑A, TMT‑B, SWT, PWT, BNT, VSRT, 5‑point, Stroop Test (NAI), Labyrinth, Interference (c.I.) are subtests of the NTBV (long version). Digit-symbol, TMT‑B, 5‑point, Stroop Test (NAI) are excluded in the NTBV (short version). SWT, PWT, VSRT are shortened in the NTBV (short version). Age and education in years

*VVT* Vienna Visuo-Constructional Test, *HC* healthy controls, *SCD* subjective cognitive decline, *MCI* mild cognitive impairment, *AD* Alzheimer’s disease, *MMSE* Mini Mental State Examination, *WST* Wortschatztest, *BDI-II* Beck Depressionsinventar II, *AKT* Alters-Konzentrations-Test, *c.I.* cerebraler Insuffizienz-Test, *TMT* Trail Making Test, *SWT* Semantischer Wortflüssigkeitstest, *PWT* Phonematischer Wortflüssigkeitstest, *BNT* Boston Naming Test, *VSRT* Verbaler Selektiver Reminding Test, *NAI* Nürnberger Altersinventar

*Correlation is significant at the adjusted alpha level of 0.001

## Discussion

We applied the present design to determine the potential of neuropsychological assessment of visuo-constructive functions using the VVT for detection of cognitive impairment and dementia. In addition, we evaluated the capacity of VVT to distinguish patients suffering from MCI and AD from the unimpaired and to classify cases correctly into these groups. To complete our analysis, we explored associations between VVT scores and other clinical variables.

### Group comparisons

We revealed significant differences in VVT scores between AD and each one of the other diagnostic groups. In other words, visuo-constructive performance was significantly lower in AD than in all other groups, confirming the value of VVT scores as indicators of visuo-constructive impairment, which is hypothesized to be strongest in this group. As expected, HC and SCD did not differ significantly since SCD patients receive this diagnosis due to perceived cognitive decline combined with inconspicuous neuropsychological performance. We expected scores to be significantly lower in MCI than in HC and SCD, but this was not the case. One possible explanation is that MCI is a heterogenous condition with different subtypes, some of which include only one cognitive domain while others include multiple domains [5], so that the degree of visuo-constructive impairment is likely to vary from one MCI case to another. Though HC showed a seemingly low average score of 73, poor performance of HC on visuo-constructive tests has previously been reported on different occasions. For instance, frequent mistakes on a pentagon-copying task and a cube-copying task have been shown to be quite prevalent in the healthy elderly [40, 41]. Thus, our finding might actually be representative of the normal variations of visuo-constructive functions in the healthy elderly population.

### Predictive validity of VVT scores for diagnostic group

The results of our ROC analysis suggested a VVT score below 70 as the best indicator for the presence of AD. Based on the diagnostic parameters computed by us using this cutoff, 78% of AD patients were identified correctly and 64% of non-AD cases were correctly classified as not having AD. Thus, 53% of cases classified as AD actually had this condition (true positives) and 85% of those classified as non-affected did not have AD (true negatives). These results show limited accuracy of VVT-based classification. The guidelines of Jaeschke et al. [42] for the interpretation of LR values would classify the VVT as having low levels of diagnostic validity; that is, the test results are unlikely to be relevant in the majority of cases but may be important in some cases. These authors also mention some confounding factors that can impact test accuracy as indicated by LR values, especially disease severity and the presence of competing diseases with similar manifestations. In fact, different manifestations of disease severity are likely in MCI, but also in AD, as patients usually live with this disease for several years after receiving the diagnosis [43]. As a result, participants with different pathology progressions and resulting varying levels of visuo-constructive impairment are likely to have been present in our sample. It should also be noted in this context that the presence of competing diseases may have impacted visuo-constructional performance differently for participants, since prior research has shown an association between the number of present comorbidities and cognitive functioning [44]. Thus, future studies assessing visuo-constructive functions should take the number of comorbid diseases into account as a possible confounding variable.

Our multinomial logistic regression analysis determined Pseudo-*R*^*2*^ at 0.27, which means that 27% of the variance of diagnostic group membership could be explained by the predictor. The classification procedures of the multinomial logistic regression model labeled all cases as either MCI or AD while HC and SCD were ignored. As a result, a low value of 53% for correctly predicted group membership was attained. In simpler terms, cases with higher VVT scores were assigned to the MCI group, which was the largest sub-sample of the non-AD groups, and those with lower VVT scores were ascribed to the AD group. The measure of agreement between predicted and observed categories was determined at a low Cohen’s Kappa of 0.21. It seems that there is not enough information in VVT scores to enable the regression model to make precise classification decisions, probably because scores are too similar in HC, SCD, and MCI, as shown also by the lack of significant differences. Thus, the main problem seems to be that the test is unable to reliably detect mild impairments present in the sample. It can, however, aid in identifying strong visuo-constructive impairment and thereby assist in distinguishing between those patients who already suffer from manifest AD and those who (so far) do not.

As proposed above, the heterogeneity of MCI might be a possible reason for this finding, and this hypothesis is supported by a recent review [45] reporting no typical order of decline across different cognitive functions in MCI, with a consequent diversity in clinical manifestations. Though the lack of significant group differences between HC and MCI clearly limits the potential of VVT scores as indicators of early dementia, these could nevertheless be used to monitor persons suffering from MCI in order to assess disease progression, as VVT scores have been shown to be significantly lower when the phase of AD is reached. Thus, future research should look into the development of VVT scores across multiple points of assessment, especially in MCI. In addition to the above, use of VVT scores as a supplemental diagnostic measure could be assessed for the identification of AD. Belleville et al. [10], for instance, report increased sensitivity when a measure of visuo-constructive functions is combined with a measure of a different cognitive function, such as associative memory. Accordingly, the VVT might also prove useful as part of a broader neuropsychological test battery, such as the NTBV.

### Correlates of VVT scores

As reported before [7], neither age, education nor premorbid IQ showed moderate or large associations with VVT scores in our sample, and the same was the case for depression. We therefore conclude that none of these variables is of relevant importance for visuo-constructive performance as measured by VVT scores. We did, however, find a moderate positive correlation revealed for VVT and MMSE scores. Since this finding is at least partly attributable to the fact that the MMSE is a screening measure for assessing the global cognitive status of patients and includes a pentagon-copying task, it should be interpreted with caution due to the compromised correlation. Previous studies [28] have shown the connection between drawing or copying performance and the global cognitive status of patients which might also explain several significant small to moderate associations that VVT scores show with many of the subtests of the broader NTBV (AKT, VSRT, Labyrinth, Interferenz etc.) that are related to distinct cognitive domains such as memory or attention. These are similar in size to those reported before by Lehrner et al. [7]. Finally, given that VVT scores did not show any large correlations with any other neuropsychological variables as measured by the NTBV, there is no problematic overlap of the measured constructs. This confirms the satisfactory discriminant validity found by the same authors.

### Strengths and limitations of this design

Some strengths and limitations should be considered for the interpretation of our findings. On the one hand, we were able to recruit a large sample including groups in several disease stages, from the impairment-free to patients with incipient conditions to those suffering from advanced deficits. On the other hand, we had to shorten the assessment for the latter group resulting in less available data regarding some of the variables of the NTBV and related correlational analyses, especially for the AD group. Furthermore, while our patients were diagnosed by experienced clinicians based on a comprehensive neuropsychological assessment, our sample only featured those patients who actively sought us out or had been referred to us. With regard to our main instrument, the VVT, additional limitations should be considered. In the first place, the fact that we conducted our analyses using raw scores of the VVT might have resulted in a confounding effect of sociodemographic variables even though correlations with VVT scores were small. Secondly, an additional instrument for the assesment of the visuo-constructive function could have served as a test of reference and provided further insight into the psychometric quality of the VVT.

In spite of these limitations, our findings will be useful for a deeper comprehension of visuo-constructive deficits in the context of dementia and have served to reveal the potential of the VVT for future research in this area.

## Footnotes

1 For a more thorough introduction to the VVT please refer to: Lehrner J. Vienna Visuo-konstruktiver Test (VVT 3.0) – ein Verfahren zur Bestimmung der Visuokonstruktion. www.psimistri.com; 2019 (Unpublished test manual). The VVT 3.0 can be obtained from www.psimistri.com.
